# Direct and Indirect Effects of Young Adults’ Relationship Status on Life Satisfaction through Loneliness and Perceived Social Support

**DOI:** 10.5334/pb.bn

**Published:** 2015-12-14

**Authors:** Katarzyna Adamczyk, Chris Segrin

**Affiliations:** 1 Institute of Psychology, Adam Mickiewicz University, ul. A. Szamarzewskiego 89/AB, 60-568 Poznań, Poland; 2 Department of Communication, University of Arizona, Tucson, AZ 85721, USA

**Keywords:** Relationship status, Life satisfaction, Loneliness, Perceived social support, Young adults

## Abstract

This study examined the indirect effects of relationship status (single *vs.* in a relationship) on life satisfaction through social and emotional (romantic and family) loneliness and perceived social support from significant others, family, and friends. Five hundred and fifty three Polish young adults (335 females and 218 males), ranging in age from 20–30 years (*M* = 23.42), completed the Polish versions of the Satisfaction With Life Scale, the Social and Emotional Loneliness Scale for Adults, and the Multidimensional Scale of Perceived Social Support. The results indicated that single individuals reported significantly lower satisfaction with life and social support from a significant other, but higher romantic and social loneliness, and higher family support compared to participants in a relationship. A path analysis revealed no direct effect of relationship status on satisfaction with life. However, there were significant indirect effects from relationship status to life satisfaction though romantic, family, and social loneliness, and through perceived social support from significant others and from family. Therefore, singlehood may be deleterious to life satisfaction because of the higher loneliness and lower social support from a significant other.

One of the fundamental human motivations is the desire for enduring intimate relationships (Spielman et al., 2013) which finds its basis in the human need for relatedness (Baumeister & Leary, 1995; Deci & Ryan, 1991). In young adulthood, a special meaning is ascribed to a close, intimate bond with a romantic partner/spouse (Erikson, 1980; Rauer, Pettit, Lansford, Bates, & Dodge, 2013). At this particular period in life individuals typically form enduring romantic relationships (Donnellan, Larsen-Rife, & Conger, 2005). The successful achievement of developmental tasks specific to young adulthood such as establishment of marriage or other long term intimate relationships is, in turn, recognized to be a crucial determinant of subjective well-being (SWB) or life satisfaction (Martikainen, 2009; Simon & Barrett, 2010). In contrast, failure to establish and sustain a committed intimate relationship during young adulthood may have serious negative implications for well-being, both concurrently and later in the life span (Kiecolt-Glaser & Newton 2001).

In light of the above, the aim of this investigation is to test the direct and indirect effects of young adults’ relationship status (single *vs*. partner relationship) on their life satisfaction through social and emotional (romantic and family) loneliness and perceived social support from significant others, family, and friends. As the linkage between involvement in different relationship types and young adults’ subjective well-being is still not fully explored (Soons & Liefbroer, 2008), this paper contributes to the ongoing discussion on the importance of relationship status for young adults’ life satisfaction. This is in part a response to the need for a more refined understanding of how relationship statuses besides marital status are related to subjective well-being (Soons & Liefbroer, 2008; Verbakel, 2012).

## Relationship Status in Polish Young Adults

The present study is performed in Poland. We consider this country to be an interesting context for research on relationship status and young adults’ life satisfaction for two main reasons. First, in Poland, trends and changes in the domain of matrimonial attitudes and behaviors are comparable to those in Western and Northern European countries. One of the important demographic transformations observed in Poland is the postponement of marriage. In Poland marriage rates have dropped off among young adults (25–29 years of age) who, until recently, had a high marriage rate. Polish census data indicate a marked increase of single women and men over just the past three years (from 27.10% to 32.80% among men, and from 19.10% to 23.90% among women). At the same time, it is worth noting that cohabitation is still much less common and less socially acceptable in Poland than in other European countries (Mynarska, Baranowska-Rataj, & Matysiak, 2014).

In addition to these changing trends in marriage, Poland is characterized by strong Catholic values and low acceptance of alternative forms of marital and family life (Baranowska-Rataj, Matysiak, & Mynarska, 2013) including singlehood and single parenthood. In Poland, the majority of people want to get married, positively value marriage, and also believe that marriage, not cohabitation, is the appropriate context for childbearing (Mynarska et al., 2014). These two above conditions create a specific social context with a rising number of single adults that is potentially at odds with the dominant pro-marriage cultural values, potentially diminishing the life satisfaction of single people. Therefore, data from Poland may provide a useful context for a test of the effects of relationship status on young adults’ life satisfaction, as it has for the happiness of Polish single mothers (see Baranowska-Rataj et al., 2013).

## Life Satisfaction and Relationship Status

Life satisfaction is the cognitive component of subjective well-being and refers to an individual’s general judgment of life conditions (Diener, Emmons, Larsen, & Griffin, 1985), to a subjective evaluation of overall quality of life (Diener & Diener, 1995). Life satisfaction is a cognitive evaluation of extent to which an individual is satisfied in life (Lucas, Diener, & Suh, 1996), and this evaluation is based on criteria deemed important by the individual (Pavot & Diener, 1993).

Traditionally marital status had been regarded as an essential determinant of various forms of well-being (Soons & Liefbroer, 2008). In general, prior studies show that married people report higher levels of well-being than people who never married, or are separated, divorced, or widowed (see Kamp Dush & Amato, 2005). Similar results were obtained in a Dutch study by Soons and Liefbroer (2008) where single young adults had the lowest level of well-being, followed by young adults who were steady dating and live together. At the same time, married young adults had the highest level of well-being (Soons & Liefbroer, 2008). Furthermore, as prior research indicated, better psychological well-being among married individuals includes both positive (i.e., life satisfaction and happiness) and negative (i.e., low depression and anxiety) aspects of well-being (Gove, Style, & Huges, 1990). Never-married individuals in turn have greater subjective well-being than previously married individuals (i.e., divorced, separated, or widowed) (Diener, Gohm, Suh, & Oishi, 2000). In a more recent study, Ben- Zur (2012) found marital status to be related to life satisfaction independently of optimism and loneliness.

## Loneliness and Relationship Status

Loneliness is a result of lacking strong, intimate bonds with significant others (Bowlby, 1973; Weiss, 1973) and it is the negative emotional response to an experienced discrepancy between the desired and actual quality or quantity of one’s relationships (Perlman & Peplau, 1981). At the same time, feeling alone or lonely does not necessarily mean being alone nor does being alone necessarily mean feeling lonely (Cacioppo, Grippo, London, Goossens, & Cacioppo, 2015).

Loneliness may be conceptualized as a multifaceted and domain-specific phenomenon. Weiss (1973) was the first to describe loneliness as a multidimensional experience and proposed a distinction between social loneliness as a result of an inadequate access to a network of peers, co-workers, neighbours, or friends, and emotional loneliness resulting from a lack of close or intimate relationships that are characteristic of ties with a romantic partner, parent, or child. Emotional loneliness is primarily related to “the absence of a partner, that is, with the absence of an exclusive, close, and intimate tie” (Dykstra & Fokkema, 2007, p. 9). In turn, social loneliness is related to a perceived deficiency in social networks, or a lack of social relations or social activities (Russell, Cutrona, Rose, & Yurko, 1984; Weiss 1973). Furthermore, on the basis of Weiss’ (1973) distinction between the experience of social isolation (social loneliness) and emotional isolation (emotional loneliness), DiTommaso and Spinner (1993, 1997) noted that emotional loneliness appeared to be comprised of two domains, that is, family emotional loneliness and romantic emotional loneliness. Loneliness is a significant predictor of life satisfaction and social well-being (Dong, Beck, & Simon, 2009; Goodwin, Cook, & Yung, 2001; Kapıkıran, 2013). It constitutes one of the factors that hinders life satisfaction (Isıklar, 2013) and a negative association between loneliness and life satisfaction has been documented in research on adolescents, university students, adults, and elderly people (see Kapıkıran, 2013).

The lack of romantic partners or intimate relationships may be an important perceived reason for one’s present feelings of loneliness (e.g., Rokach & Brock, 1998). For example, married individuals and individuals living with a significant other reported less romantic loneliness than those who were not in such relationships (Bernardon, Babb, Hakim-Larson, & Gragg, 2011). DiTommaso and Spinner (1993) revealed that being involved in a romantic relationship was significantly related to lower levels of romantic loneliness, although it was only weakly linked to family and social loneliness. Similarly, Çeçen (2007) found that not being involved in a romantic relationship was related to great romantic loneliness, but not with family or social loneliness. Furthermore, a study of Polish young adults aged 19–25 revealed that single participants reported greater levels of romantic and family loneliness, although they did not show lower social loneliness than participants who were involved in a relationship (Adamczyk, 2015).

## Perceived Social Support and Relationship Status

A positive association between social support and life satisfaction has been evidenced in numerous prior studies, even when controlling for covariates such as personality (Kong & You, 2013). Perceived social support refers to perceptions of the extent to which people from one’s social network are available to provide support (e.g., Demaray & Malecki, 2002). Social support is considered to be an important factor in mental health and well-being (e.g., Segrin & Domschke, 2011; Zimet, 1998). Some authors have also suggested that perceived social support may have a more significant effect on problems such as loneliness than actual received social support (e.g., Pinquart & Sorensen, 2003).

Involvement in a romantic relationship is predictive of higher perceived social support. Several studies have shown that married individuals reported significantly greater support from their significant other than single individuals did, but no significant differences were found between the two groups on perceived social support from family and friends (Prezza & Pacilli, 2002; Zimet, Powell, Farley, Werkman, and Berkoff, 1990). Also, in a Polish study (Adamczyk, 2015), single participants reported lower levels of perceived social support from family and significant others compared to partnered counterparts, but no differences emerged in regard to perceived social support from friends.

## The Current Study

The present study is part of a larger research project concerning subjective well-being and mental health among single young adults and young adults in non-marital romantic relationships in Poland (Adamczyk & Segrin, 2014). In another paper (Adamczyk & Segrin, 2014) we already presented results concerning mental health reported by single young adults compared to those in non-marital romantic relationships.

In light of prior findings of differences between single and partnered individuals and the theoretical affordances of such relationships, the following hypotheses were formulated:

H1: Single young adults will report lower life satisfaction than young adults in non-marital romantic relationships.

H2: Single young adults will report higher romantic loneliness than young adults in non-marital romantic relationships.

H3: Single young adults will report lower social support from significant others than young adults in non-marital romantic relationships.

What is less clear from prior research is whether relationship status (i.e., single versus in a relationship) is uniquely associated with deficits in social support from significant others and the emotional loneliness that results from the lack of an attachment figure, or alternatively, if relationship status is a proxy for a more generalized social relationship deficit that could be associated with more pervasive deficits in social support and loneliness in a wide range of contexts. We therefore sought to test the following research questions:

RQ1: Will single young adults report similar family and social loneliness as young adults in non-marital romantic relationships?

RQ2: Will single young adults report similar perceived social support from family and from friends as young adults in non-marital romantic relationships?

Finally it was predicted that:

H4. Loneliness and perceived social support will mediate the association between relationship status and life satisfaction.

## Method

### Participants and Procedure

Participants in this study were 553 young adults who were a mix of non-students and university students from different faculties at Adam Mickiewicz University in Poznań, Poland. All participants resided in Poznań, a large Polish city with a population exceeding 500,000. All the respondents were heterosexual, never married, and had no children, and declared that they wanted to have a lifetime partner in the future. One thousand questionnaires were originally distributed. A total of 688 participants returned questionnaires (response rate = 68.80%). Of these, 135 participants were removed because they were married, widowed, divorced, separated, were involved in a relationship for a period shorter than 6 months, or because of incomplete data, yielding a final sample of 553 participants. The university students constituted 48.80% of the total sample (*n* = 270), while non-student participants constituted 51.20% of the total sample (*n* = 283). The age of participants ranged from 20 to 30 years old (*M* = 23.42, *SD* = 3.27). Women represented 60.60% of the sample (*n* = 335) and men represented 39.40% of the sample (*n* = 218).

Two hundred and seven participants (37.43%) reported being single at the time of the assessment, whereas 346 participants (62.57%) had a non-marital romantic partner. Being single was defined for participants as “not in a committed relationship for at least 6 or more months, but wanting to become committed in the near future (within the next year or so)”, and being in a non-marital romantic relationship was defined as “in a non-marital romantic relationship (for at least 6 or more months), but wanting to become committed in the near future (within the next year or so)” (see Schachner, Shaver, & Gillath, 2008). The criterion of 6 months as qualification for involvement in a relationship was based on a prior study performed by Donnelly and Burgess (2008). Regarding this criterion, all participants who were not single but were in a nonmarital romantic relationship for a period shorter than 6 months were excluded from further analysis.

The first author distributed questionnaires to students during different classes (to groups of 20 to 30 students ) with the request to administer the questionnaires to their social network members, but not to distribute the survey to partners in the same relationship in order to avoid violation of the assumption of independence. The purpose of the study was explained along with assurance to students that all information provided would remain anonymous. Then, if they choose to participate, their consent was implicitly understood. We adhered to institutional ethical guidelines in the conduct of this study (i.e., guidelines of American Psychological Association and Polish Psychological Society were followed in the treatment of participants).

## Measures

The questionnaire package presented to the study participants was comprised of the following instruments.

**Demographic Questionnaire**. This questionnaire was designed to obtain general descriptive information about participants’ background such as their age, sex, education, and current relationship status.

**Multidimensional Scale of Perceived Social Support** (MSPSS; Zimet et al., 1988) (Polish adaptation by Adamczyk, 2013). The MSPSS is a self-report instrument that measures the adequacy of one’s perceived social support from three domains: family, friends, and a significant other. The first scale refers to perceived social support from family (e.g., *“My family really tries to help me.”*). The second scale includes items concerning perceived social support from friends (e.g., *“I have friends with whom I can share my joys and sorrows.”*). The third scale refers to perceived social support from significant others (e.g., *“There is a special person who is around when I am in need.”*). There are four items per subscale, each with response options ranging from 1 (*very strongly disagree*) to 7 (*very strongly agree*). Higher scores on each of the subscales indicate higher levels of perceived support. Zimet et al. (1988) investigated and found internal reliability estimates of .93, .92, .93 for Friends, Family, and Significant Other subscales, respectively. In the present study internal consistency for the subscales was very high at α = .94, α = .91, α = .95 for the Significant Other, Family, and Friends Support scales, respectively.

**Satisfaction With Life Scale** (SWLS; Diener et al., 1985) (Polish adaptation by Juczyński, 2009). The SWLS is a 5-item instrument designed to measure global cognitive judgments of satisfaction with one’s life. The SWLS uses a 7-point Likert scale, ranging from 1 (*strongly disagree*) to 7 (*strongly agree*), yielding a possible range of 5–35. Past research has shown that the scale’s internal consistency was high (α = .87) and two week test-retest reliability was *r* = .85 (Diener et al., 1985). The Cronbach’s alpha in the current study was .85.

**The Social and Emotional Loneliness Scale for Adults – Short Form** (SELSA-S; DiTommaso et al., 2004) (Polish adaptation by Adamczyk & DiTommaso, 2014). The SELSA-S is a multidimensional measure of loneliness that consists of 15 items rated on a 7-point Likert-type scale, ranging from 1 (*strongly disagree*) to 7 (*strongly agree*). It was designed to measure emotional (romantic and family) and social loneliness. Each subscale consists of five statements about feelings of loneliness within the past year. The family loneliness subscale assesses feelings toward family relationships (e.g., *“My family really cares about me.”*). The social loneliness subscale measures feelings toward being part of a social group (e.g., *“My friends understand my motives and reasoning.”*). The romantic loneliness subscale measures the degree to which participants feel they have social support from a significant other (e.g., *“I have a romantic or marital partner who gives me the support and encouragement I need.”*). The SELSA-S’s three subscales have high internal reliability, with Cronbach’s alpha coefficients ranging from .87 to .90, and have been shown to be valid measures of loneliness (DiTommaso et al., 2004; DiTommaso et al., 2007). In the present study, Cronbach’s alpha coefficients were: .83 (romantic), .87 (family), and .84 (social).

## Data Analysis

**Figure 1:**
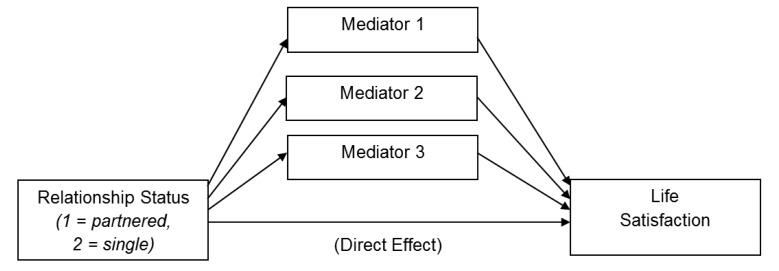
Conceptual Depiction of Parallel Mediation Models.

To test the hypotheses (H1, H2 and H3) and research questions (RQ1 and RQ2) concerning the possible differences between single individuals and individuals in non-marital romantic relationships with regard to life satisfaction, loneliness, and perceived social support, one-way analyses of variance (ANOVA) and one-way multivariate analyses of variance (MANOVA) were performed (see Table 1). Next, to evaluate the fourth hypothesis about the indirect effects, associations between relationship status and life satisfaction, through loneliness (romantic, family, and social) and perceived social support (from significant others, family and friends), were tested in the PROCESS module within SPSS 22. This is a regression-based path analysis program. For these analyses, a parallel mediation model was specified and a bias corrected bootstrapping procedure based on 5000 bootstrap samples was used to estimate standard errors around the indirect effects. Two such models were analyzed. For all models, relationship status, dummy coded as 1 = partnered, 2 = single, was the independent variable and life satisfaction was the dependent variable. For the first model, the three forms of loneliness were specified as parallel mediators. In the second model, the three forms of perceived social support were specified as the parallel mediators. For the purpose of illustration, a conceptual model of these parallel mediation analyses appears in Figure 1.

**Table 1:** Means and Standard Deviations of Life Satisfaction, Loneliness and Perceived Social Support by Relationship Status.

	Total sample (N = 553)	Single sample (N = 207)	Partnered sample (N =346)	*F value*	*η^2^*

Variables	Mean (SD)	Mean (SD)	Mean (SD)		
Satisfaction with life	21.68 (5.68)	20.69 (5.54)	22.27 (5.70)	10.25**	.02
Multivariate test				190.05***	.51
*Loneliness*					
Romantic loneliness	13.61 (8.18)	21.01 (6.14)	9.19 (5.66)	530.48***	.49
Family loneliness	10.61 (5.39)	11.18 (5.35)	10.27 (5.39)	3.74	.01
Social loneliness	11.21 (5.18)	10.60 (4.67)	11.57 (5.44)	4.58*	.01
Multivariate test				57.28***	.24
*Perceived social support*					
Significant other support	19.19 (4.41)	16.84 (4.89)	20.60 (3.40)	113.26***	.17
Family support	17.43 (4.46)	16.94 (4.55)	17.72 (4.38)	3.99*	.01
Friends support	17.67 (4.11)	17.76 (4.02)	17.62 (4.18)	.14	.00

*Note*. * *p* < .05; ** *p* < .01; *** *p* < .001.

For clarity of presentation, the results are presented separately in four major sections. The first, second, and third sections present results concerning differences between single and partnered individuals with regard to life satisfaction, loneliness, and perceived social support. The fourth section presents the direct and indirect effects of young adults’ relationship status on life satisfaction through loneliness and perceived social support.

## Results

### Life Satisfaction and Relationship Status

In order to examine the mean differences in life satisfaction between single individuals and individuals in non-marital romantic relationships, a one-way ANOVA was performed. The analysis indicated a significant effect of relationship status on life satisfaction, *F*(1, 552) = 10.25, *p* < .001, η*^2^* = .02, such that single individuals scored lower on life satisfaction than did individuals in non-marital romantic relationships (see Table 1).

### Loneliness and Relationship Status

Mean differences in loneliness between single individuals and individuals in non-marital romantic relationships were tested with a one-way multivariate analysis of variance (MANOVA) treating each of the three loneliness scales as a dependent variable. The analysis revealed a significant multivariate effect of relationship status on loneliness, Wilks’s Λ = .49, *F*(3, 549) = 190.05, *p* < .001, *η^2^* = .51. Follow-up univariate analyses revealed that single individuals scored higher on romantic loneliness and lower on social loneliness than did individuals in non-marital romantic relationships (see Table 1). At the same time, no differences emerged between single individuals and individuals in non-marital romantic relationships in the domain of family loneliness.

### Perceived Social Support and Relationship Status

Potential differences in social support among single individuals and individuals in non-marital romantic relationships were tested with a multivariate analysis of variance, treating the three social support subscales as dependent variables. The analysis revealed a significant multivariate effect of relationship status on perceived social support, Wilks’s Λ = .76, *F*(3, 549) = 57.28, *p* < .001, *η^2^* = .24. Follow-up univariate analyses (see Table 1) revealed that single individuals scored significantly lower on perceived social support from significant others and lower on perceived social support from family compared to individuals in non-marital romantic relationships. At the same time, no differences emerged between the two groups for perceived social support from friends.

### Direct And Indirect Effects of Young Adults’ Relationship Status on Life Satisfaction through Loneliness and Perceived Social Support

The first model estimated the direct effect of relationship status on life satisfaction through loneliness. This model simultaneously estimated the indirect effects through the three different forms of loneliness assessed in this investigation: romantic, family, and social loneliness. The results of this analysis are presented in the upper half of Table 2 and revealed a total effect of relationship status on life satisfaction (*B* = −1.59, *p* = .001). The direction of this effect would imply that those participants who were in a nonmarital romantic relationship reported higher life satisfaction than those participants who were single. However, it is clear that this total effect is mostly due to the indirect effects as the direct effect of partner status on life satisfaction was not statistically significant.

**Table 2:** Total, Direct, and Indirect Effects of Relationship Status on Life Satisfaction through Parallel Mediation Models for Loneliness and Perceived Social Support.

Total effect	Direct effect	Total Indirect effect	Individual Indirect Effects	R2 for Model

**Loneliness Model**				

−1.59** (0.50)	0.26 (.063)	−1.84*** (0.54), CI_95_ = [−3.00, −.086]	Romantic loneliness: −1.79*** (0.50), CI_95_ = [−2.89, −0.90] Family loneliness: −.27* (0.14), CI_95_ = [−0.58, −0.02] Social loneliness: .22* (0.11), CI_95_ = [−0.04, −0.49]	.23***
**Perceived Social Support Model**				

−1.59** (0.50)	−0.49 (.063)	−1.09*** (0.34), CI_95_ = [−1.78, −0.47]	Significant Other support: −0.82** (.27), CI_95_ = [−1.35, −0.28] Family support: −0.29* (.15), CI_95_ = [−0.63, −0.02] Friend support: 0.01 (0.04), CI_95_ = [−0.04, 0.14]	.20***

*Note.* Table values are unstandardized regression coefficient. Values between brackets are standardized errors. * *p* < .05, ** *p* < .01, *** *p* < .001.

The results in Table 2 show that there was a significant total indirect effect of relationship status on higher life satisfaction through lower loneliness (*B* = −1.84, *p* < .001). This total effect can be deconstructed through analysis of each of the three types of loneliness. The results showed that the indirect effects through romantic, family, and social loneliness were all statistically significant (see Table 2). It should be noted that the direction of the effect for social loneliness indicates that partnered participants had higher social loneliness than their single peers. Therefore, these findings show that people in non-marital romantic relationships reported significantly lower romantic and family loneliness, and this at least partially explained why those in non-marital romantic relationships experience higher life satisfaction. Collectively, these direct and indirect effects explained 23% of the variance in life satisfaction.

The second model also estimated the direct and indirect effects between relationship status and life satisfaction, this time including the three forms of perceived social support as parallel mediators. As indicated in the lower half of Table 2, the total indirect effect of relationship status on life satisfaction, thought perceived social support, was statistically significant, *B* = −1.09, *p* < .001. A deconstruction of this indirect effect by its three individual components revealed a significant indirect effect through perceived social support from significant others and family, but not through perceived social support from friends. These effects indicate that part of the reason why relationship status has an effect on life satisfaction is because single people report significantly lower perceived social support from family and significant others than people in non-marital romantic relationships do. However, there was no such indirect effect through perceived social support from friends, because in this model, relationship status was not related to perceived social support from friends.

Collectively, these direct and indirect effects explained 20% of the variance in life satisfaction.

## Discussion

This study was designed to determine the extent to which social and emotional (i.e., romantic and family) loneliness and perceived social support from three distinct sources (i.e., significant others, family, and friends) explain the association between relationship status and life satisfaction among young adults in Poland. Our analysis concerning the differences between single and individuals in a relationship with regard to life satisfaction, loneliness, and perceived social support revealed that young adults in non-marital romantic relationships experienced higher life satisfaction than single young adults. Individuals in non-marital romantic relationships also reported lower romantic loneliness, but higher social loneliness in comparison to single individuals. Furthermore, people in relationships evinced of higher perceived social support from significant others and family. Moreover, our results showed that the association between relationship status and life satisfaction is attributable to indirect effects through various forms of loneliness and social support.

Our results corroborate prior studies showing that married individuals experience higher life satisfaction than previously married individuals, with never-married persons in an intermediate position (Gove et al., 1983). In general, prior research indicated the highest level of subjective well-being among married individuals (e.g., Kamp Dush & Amato, 2005; Verbakel, 2012). The lowest level of well-being was found among single people, followed by dating, cohabiting, and finally married young adults (Soons & Liefbroer, 2008). The lower level of life satisfaction among single individuals may reflect the fact that establishing and maintaining romantic relationships constitutes a fundamental developmental activity in young adults’ lives (Erikson, 1980). Therefore, if single individuals expect and value commitment to a romantic relationship, this mismatch between their expectations and actual life situation (i.e., being single) may result in lower life satisfaction than those in romantic relationships who have met these expectation and needs.

Single people understandably reported higher romantic loneliness than people in a non-marital romantic relationship. Past studies have shown that married individuals and individuals living with a significant other experience less romantic loneliness than those who are not in such relationships (e.g., Adamczyk, 2015; Bernandon et al., 2011; Çeçen, 2007; DiTommaso & Spinner, 1993). In contrast, the lack of a romantic partner is associated with a type of loneliness specific to lack of an attachment figure (Dykstra & Fokkema, 2007; Rokach & Brock, 1998).

With respect to the perceived social support from significant others, it appears that the lack of a significant other is a major determinant to feeling supported by a particular and important other person in one’s life as would be experienced by a married individual for example (Adamczyk, 2015; Prezza & Pacilli, 2002; Zimet et al., 1990). Significant others (e.g., a romantic partner) are often around when an individual is in need and share joys and sorrows. Lack of this type of partner creates obvious deficits in this specific form of social support.

In the current study, we proposed several research questions concerning the association between relationship status and perceived social support as well as loneliness in domains other than romantic partners and significant others. Although one could easily anticipate perceived deficits in social support from significant others and romantic loneliness among single individuals, it was unclear if these negative associations with singlehood would carry over to other social relationships. If relationship status could be viewed as a proxy for a more generalized social relational deficit, then one might expect loneliness and lack of social support in other contexts as well. However, if singlehood is a marker of a social standing that is unique to romantic relationships, there is no reason to expect a carry over to deficits in other social contexts.

The results of this study provided only minimal evidence that singlehood may be an indicator of a more pervasive deficit in social relationships. First, our analyses indicated no significant differences in family loneliness as a function of relationship status, and only slight differences in social loneliness, with partnered individuals reporting slightly higher social loneliness than participants in non-marital romantic relationships. Interestingly, the effect for social loneliness was such that partnered individuals reported slightly higher social loneliness than single individuals. Second, social support from family, but not friends, was lower among single participants compared to partnered participants. However, this difference was weak in magnitude. Based on the correlational analysis we can conclude that singlehood does not represent a global deficit in social relationships, but basically is associated with deficits that are specifically consequential to lacking a romantic partner. This conclusion is congruent with some prior studies that indicated that not being involved in a romantic relationship was related to higher scores on the romantic loneliness scale, but not with scores on the family or social loneliness scales (Çeçen, 2007).

Finally and most importantly, in the current study we predicted that relationship status would have an indirect effect on life satisfaction through various forms of loneliness and social support (H4). Pursuant to that hypothesis, we tested two parallel mediation models in which three forms of loneliness and three forms of perceived social support were separately tested as mediators. The results of this study not only indicated bivariate associations between young adults’ relationship and life satisfaction, but add to the literature on partner status and well-being by documenting indirect effects through social support and loneliness. In particular, our findings revealed that higher life satisfaction among individuals in non-marital romantic relationships can be explained in part by the lower loneliness experienced by people with a partner. To be precise, higher life satisfaction among individuals in non-marital romantic relationships can be explain by lower romantic and family loneliness. In addition, although people in non-marital romantic relationships showed lower romantic and family loneliness which leads to higher life satisfaction, they also showed slightly higher social loneliness, which in itself has a negative effect on life satisfaction.

It should be pointed out that our findings corroborate those of other investigators who found that loneliness is negatively related to life satisfaction (e.g., Goodwin et al., 2001; Isıklar, 2013; Kapıkıran, 2013). Our findings strongly indicated that social, romantic, and family loneliness are antagonistic to young adults’ life satisfaction. Furthermore, an unexpected finding merits special attention. Single participants reported lower social loneliness than those in non-marital romantic relationships. Social loneliness primarily refers to unmet needs in the wider network of support givers (Dykstra & Fokkema, 2007). Thus it appears that those in a committed relationship actually experience these unmet needs to a greater extent than those who are single. This may be due to a displacement effect whereby people with a partner heavily invest time and energy in their romantic relationships, to the exclusion of and cost to other types of relationships. Supporting this assumption, Barrett’s (1999) study revealed that never married participants aged 30-45 had more frequent interaction with friends and relatives than their currently or previously married peers. Results of the current study suggest that primary romantic attachment may displace opportunities for interaction with a network of peers, co-workers, neighbours, or friends (Weiss, 1973), culminating in higher social loneliness among partners than among single people. This tempers and complicates conclusions about the social benefits of being in a partnered relationship for young adults. Such relationships may protect against feelings of emotional loneliness, but that protection may come at the cost of higher social loneliness.

Our findings revealed that lower life satisfaction among single versus partnered individuals can be explained in part by their lower perceived social support from significant others and family, but not from friends. The importance of significant others (e.g., romantic partners) and family to life satisfaction in young adulthood could be understood in the context of adult attachment. Young adulthood is a period in life when romantic partners become primary attachment figures, and in the case of the lack of a lifetime partner – as it happens among single individuals – parents and siblings may still be the main attachment figures (Doherty & Feeney, 2004), and serve as an essential source of support in the absence of a romantic partner. This suggests that perceptions of support from family and significant others may contribute to higher life satisfaction among indviduals in non-marital romantic relationships.

Perhaps the most compelling indicator of a more pervasive social deficit associated with singlehood comes from the finding that single people reported lower social support from family than partnered people did. It seems probable that despite the significance of family relationships in single individuals’ lives, they may perceive these relationships and support from family as non-sufficient and inadequate to their needs and desires. This may contribute to a negative emotional response to percived deficits in the quality or quantity of one’s relationships with family members.

## Limitations and Future Directions

This study has a number of limitations that could be addressed in future research. First, the study design was cross-sectional. Therefore, it is impossible to draw definitive causal inferences about relationship status on other variables in the investigation. In the future it would be useful to replicate these findings with a cross-lagged design because dissatisfaction with life may lead people to withdrawal from romantic, family, and social relationships contributing to greater loneliness and less social support (cf. Mellor, Stokes, Firth, Hayashi, & Cummins, 2008). Second, it would be useful for future research to assess attitudes towards solitude and singlehood as prior research on adolescents’ aloneness revealed that aversion and affinity towards solitude are associated with different coping strategies with loneliness and different psychological adjustment (Teppers, Luyckx, Vanhalst, Klimstra, & Goossens, 2014). Third, testing these models in more diverse samples would also be useful in future research. It will be useful focus future research on divorced, widowed, and separated individuals as there is great diversity within the group of people categorized as “single.” It would be equally important to study well-being as a function of relationship status in non-heterosexual populations. Fourth, this study only measured inclusion in a romantic relationship, not relationship quality. Assessing relationship quality has high potential to enhance explanation of life satisfaction (Kamp Dush & Amato, 2005) and may mediate the effects of relationship status on well-being (Soons & Liefbroer, 2008). Finally, it would be valuable to replicate these findings with non-Polish individuals to make cross-cultural comparisons. The Polish culture is heavily influenced by the Roman Catholic religion, which affects social norms and attitudes concerning family formation, and the level of social disapproval of alternative marital and family forms (Baranowska-Rataj et al., 2013) such as single living.

Notwithstanding the aforementioned limitations, the current study extends insight into underlying mechanisms between relationship status and life satisfaction in a sample of Polish young adults. The research findings provide explanations as to why people in relationships enjoy greater life satisfaction than those who are single. Namely, low levels of loneliness and high levels of social support at least partially account for this relationship. The present study highlights the role of several theoretically important intervening constructs in this association. The results suggest that relationship status affects life satisfaction through social and emotional (i.e., romantic and family) loneliness. Moreover, this study showed that remaining single may be associated with deficits in some relationships (i.e., romantic and family), but benefits to others (i.e., friends). At the same time, single status appears to have social implications the reach beyond just romantic relationships. One practical implication of these results would be provision of counseling services for distressed single young adults that focus on ameliorating loneliness in addition to identifying and enhancing access to various sources of social support in order to improve life satisfaction.

## Competing Interests

The authors declare that they have no competing interests.
